# Tissue-resident memory T cells contribute to protection against heterologous SARS-CoV-2 challenge

**DOI:** 10.1172/jci.insight.184074

**Published:** 2024-12-06

**Authors:** Abby Odle, Meenakshi Kar, Abhishek K. Verma, Alan Sariol, David K. Meyerholz, Mehul S. Suthar, Lok-Yin Roy Wong, Stanley Perlman

**Affiliations:** 1 Department of Microbiology and Immunology, University of Iowa, Iowa City, Iowa, USA.; 2 Center for Childhood Infections and Vaccines of Children’s Healthcare of Atlanta, Department of Pediatrics, Emory University School of Medicine, Atlanta, Georgia, USA.; 3 Emory Vaccine Center, Atlanta, Georgia, USA.; 4 Department of Medicine, Washington University School of Medicine, St. Louis, Missouri, USA.; 5 Department of Pathology, University of Iowa, Iowa City, Iowa, USA.; 6 Department of Microbiology and Immunology, Emory University School of Medicine, Atlanta, Georgia, USA.; 7 Department of Microbiology, Biochemistry and Molecular Genetics and; 8 Center for Virus-Host-Innate Immunity, Rutgers New Jersey Medical School, Newark, New Jersey, USA.

**Keywords:** COVID-19, Virology, Adaptive immunity, T cells

## Abstract

New vaccine formulations are based on circulating strains of virus, which have tended to evolve to more readily transmit human to human and to evade the neutralizing antibody response. An assumption of this approach is that ancestral strains of virus will not recur. Recurrence of these strains could be a problem for individuals not previously exposed to ancestral spike protein. Here, we addressed this by infecting mice with recent SARS-CoV-2 variants and then challenging them with a highly pathogenic mouse-adapted virus closely related to the ancestral Wuhan-1 strain (SARS2-N501Y_MA30_). We found that challenged mice were protected from severe disease, despite having low or no neutralizing antibodies against SARS2-N501Y_MA30_. T cell depletion from previously infected mice did not diminish infection against clinical disease, although it resulted in delayed virus clearance in the nasal turbinate and, in some cases, in the lungs. Levels of tissue-resident memory T cells were significantly elevated in the nasal turbinate of previously infected mice compared with that of naive mice. However, this phenotype was not seen in lung tissues. Together, these results indicate that the immune response to newly circulating variants afforded protection against reinfection with the ancestral virus that was in part T cell based.

## Abstract 2

Mice infected with Omicron strains of SARS-CoV-2 are protected against challenge with virulent mouse-adapted ancestral virus in the absence of neutralizing antibody.

## Introduction

SARS-CoV-2, the etiological agent of COVID-19, has evolved repeatedly since it first entered human populations in late 2019 (1, 2). Although the virus grew well in the human upper and lower respiratory tract at the time of its initial introduction, virus evolution took place over the next several months to enhance replication and transmission to susceptible individuals. Effective vaccines became widely available in December 2020. Vaccination, in conjunction with widespread infection, resulted in widespread immunity and selection for mutations in the virus that evaded the antivirus antibody response, in addition to enhancing virus replication. Many of these variants contained greater than 20 mutations in the S protein (3, 4), leading to the hypothesis that evolution had occurred in a persistently infected immunocompromised host (5–7), although this has not been proven.

Vaccines were formulated originally to induce a neutralizing antibody response against the ancestral virus strain (Wuhan-1), and studies in 2020–2021 showed that neutralizing antibody titers served as useful correlates of protection (8). As the virus has mutated, effective neutralizing antibody titers against the newest variants elicited by ancestral virus vaccines diminished several hundred–fold, so that they are often undetectable (9–11). At the same time, serum neutralizing antibody titers against Wuhan-1 declined, suggestive of a lack of durability (12, 13). Yet, even with this loss of the ability to neutralize the virus, humans are largely protected from hospitalization and death. These results suggest that other arms of the immune response, including the antivirus T cell response; Fc receptor–dependent, nonneutralizing antibody function; or the innate myeloid response contribute to protection (3, 14, 15).

At present, vaccine strategies involved eliciting a neutralizing antibody response against the circulating strain of SARS-CoV-2. For a short period of time, vaccines were bivalent and contained spike proteins from the Wuhan-1 and circulating (Omicron BA.1 or BA.5) strains. However, inclusion of the ancestral S protein induced a strong anamnestic immune response to antibody epitopes present in this strain, some of which were no longer expressed in the Omicron strains. Consequently, the ancestral S protein was removed from the latest vaccine formulations, which express a single Omicron S protein (XBB.1.5 JN.1 or KP.2). While there is little evidence that ancestral-like variants that formerly circulated in human populations have resurged, there is a concern that reemergence of these strains would cause significant disease in populations that have only been infected with or vaccinated against more recent variants. Since SARS-CoV-2 has only been circulating briefly in human populations as compared with other viruses, the trajectory for its evolution is not well understood. Moreover, it has been reported that immunocompromised patients are persistently infected by SARS-CoV-2 and harbor mutated virus (16). The presence of diverse SARS-CoV-2 species poses a risk for reemergence of ancestral-like variants.

To examine this possibility, we infected mice with Omicron variants and then challenged them with earlier strains. Unlike ancestral strains, nearly all of the earlier strains, apart from the B.1.617 (δ variant), can infect laboratory mice directly, but disease is mild. Therefore, the effects of prior infection or vaccination are usually addressed by assessing virus titers in the lungs or nasal cavity (17, 18). In addition to measuring virus titers, we challenged mice with a mouse-adapted virus derived from the Wuhan-1 strain (SARS2-N501Y_MA30_) by repeated passage through mouse lungs (19). Mouse adaptation involved 5 changes in the spike protein and 3 in nonstructural proteins. Of the 5 S protein mutations, 4 of the 5 were present in all Omicron strains (K417N, E484K, Q498R, and N501Y), whereas the fifth mutation, Q493R, was present in a subset of Omicron variants. Moderate doses of SARS2-N501Y_MA30_ cause lethal disease in BALB/c mice of all ages and in C57BL/6 mice greater than 3 months of age (19). Using mice initially infected with SARS-CoV-2 variants, we found complete protection against clinical disease after challenge with SARS2-N501Y_MA30_ in the absence of any detectable neutralizing antibody. These results were confirmed using a virus reduction assay after challenge with the B.1.1.7 (α) variant, which was present in human populations early in the pandemic and is able to infect mice directly. SARS-CoV-2–specific T cells provided part but not all of the protection afforded by prior infection. Thus, prior infection with a recently circulating Omicron strain resulted in protection against challenge with variants no longer in human populations, with protection conferred by antivirus T cells as well as immune mechanisms that require further characterization.

## Results

### SARS-CoV-2 variants induce a neutralizing antibody response that wanes over time.

**Figure 1:**
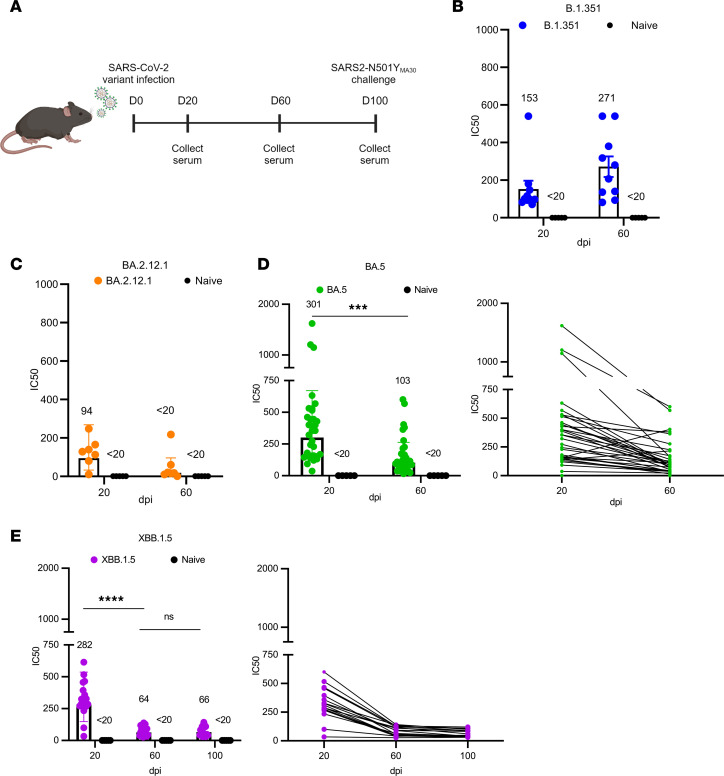
Neutralization against SARS-CoV-2 variants. Mouse neutralizing antibody titers against SARS-CoV-2 variants were measured over time using an FRNT_50_ assay. (**A**) Schematic of intranasal mouse infection and sera collection. (**A**–**E**) Variants included were (**B**) B.1.351, (**C**) BA.2.12.1, (**D**) BA.5, and (**E**) XBB.1.5. Naive mice were uninfected. (**B**) B.1.351 (*n* = 10) and naive (*n* = 5). (**C**) BA.2.12.1 (*n* = 7) and naive (*n* = 5). (**D**) BA.5 (*n* = 32) and naive (*n* = 5). (**E**) XBB.1.5 (*n* = 19) and naive (*n* = 5). Antibody titers were determined by the highest antibody dilution that resulted in a 50% reduction in the number of foci. 50% serum neutralization titers (NT_50_) are listed above each group. Limit of detection (LOD) = 20 PFU. *P* values were measured by 1-way ANOVA followed by Tukey’s test for multiple comparisons. ***P < 0.001, ****P < 0.0001.

Several previous studies reported the waning of SARS-CoV-2–specific neutralizing antibody titers following vaccination or infection and significantly decreased neutralizing activity against new variants of concern (20–23). We sought to not only evaluate the kinetics of the antibody response elicited by circulating variants after infection, but also if these presently circulating strains afforded protection against SARS-CoV-2 strains present early in the pandemic. Our study was divided into 4 cohorts of C57BL/6 mice that were intranasally infected with high doses of B.1.351, BA.2.12.1, BA.5, or XBB.1.5 virus (Figure 1A). Sera were collected from mice at various days postinfection (dpi), and titers against the homologous virus were measured. The neutralizing titers in each cohort against the corresponding variant, except those against B.1.351, were significantly reduced over time. B.1.351 is known to be more virulent in mice than other human strains, contributing to the enhanced immune response (24–26). The reduction was most pronounced for most variants between 20 and 60 dpi (Figure 1, C–E). In contrast, B.1.351-infected mice showed modest increase in antibody response from 20 to 60 dpi (Figure 1B). Mice previously infected with BA.2.12.1 exhibited lower levels of neutralizing antibodies at 20 dpi relative to the other variants and had minimal neutralizing activity by 60 dpi (Figure 1C). Mice infected with BA.5 or XBB.1.5 had similar antibody levels, with 50% serum neutralization titers (NT_50_) of 301 and 282, respectively, at 20 dpi (Figure 1, D and E, left). Individual titers for each mouse were tracked over the course of the experiment. We found that XBB.1.5 titers waned from 20 to 60 days, with minimal changes in titers from 60 to 100 days (Figure 1E, right). These data indicated that each variant was able to induce a neutralizing antibody response to the homologous virus, but peak neutralizing titers varied greatly by variant. Additionally, there was evidence of significant waning in neutralizing antibodies for each virus, apart from B.1.351, just 2 months after infection. However, the rate of waning was much less pronounced 3+ months later, consistent with patterns of SARS-CoV-2 antibody decline described previously (27).

### Infected mice are protected from reinfection with lethal SARS2-N501Y_MA30_.

**Figure 2:**
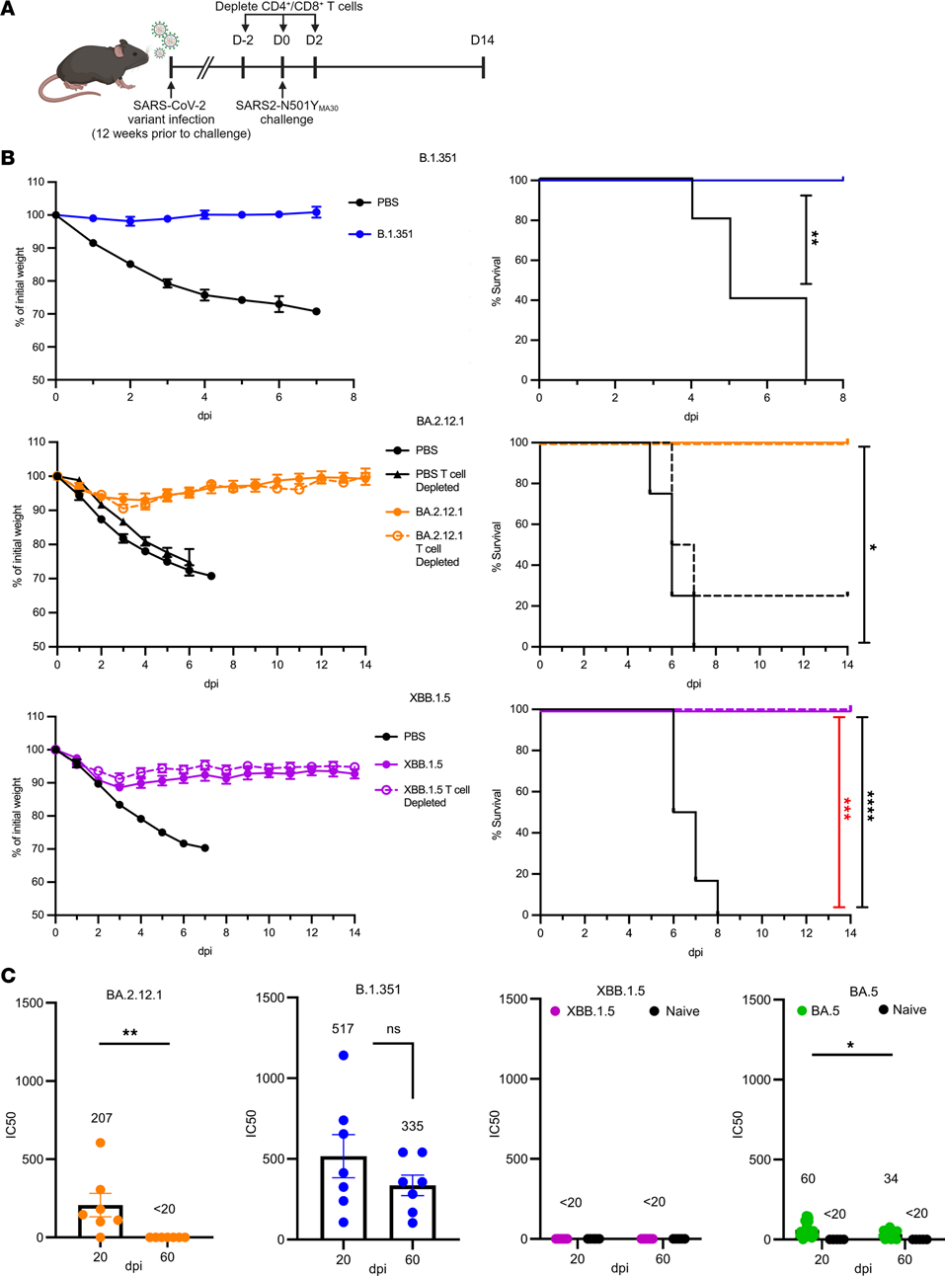
Sequential infection of mice with SARS-CoV-2 variants of concerns followed by SARS2-N501Y_MA30_. (**A**) Four-month-old (XBB.1.5) or 6-month-old (other SARS-CoV-2 variants) C57BL/6 mice were infected intranasally with SARS-CoV-2 variants or mock infected and were challenged with a lethal dose of SARS2-N501Y_MA30_ 3 months later. (**B**) Mice previously infected with B.1.351 (blue), BA.2.12.1 (orange), or XBB.1.5 (purple) or mock infected (PBS) were assessed for weight loss and survival after SARS2-N501Y_MA30_ challenge. In some experiments, T cells were depleted at the time of challenge (depleted). *B.1.351* data are from 1 experiment. PBS (*n* = 5) and B.1.351 (*n* = 4). *BA.2.12.1* data are from 2 independent experiments. PBS (*n* = 9), PBS depleted (*n* = 4), BA.2.12.1 (*n* = 7), and BA.2.12.1 depleted (*n* = 5). *XBB.1.5* data are from 2 independent experiments. PBS (*n* = 6), XBB.1.5 (*n* = 8), and XBB.1.5 depleted (*n* = 7). Red statistics denote PBS vs. XBB.1.5 depleted, black statistics denote PBS vs. XBB.1.5. *P* values were measured by log-rank followed by Bonferroni’s correction for multiple comparisons. (**C**) Sera obtained prior to challenge were tested for SARS2-N501Y_MA30_ neutralizing antibodies. B.1.351 (blue), BA.2.12.1 (orange), BA.5 (green) or XBB.1.5 (purple), B.1.351 (*n* = 7), BA.2.12.1 (*n* = 7), BA.5 (*n* = 32), XBB.1.5 (*n* = 19), and naive (*n* = 5/group). Antibody titers were determined by the highest antibody dilution that results in a 50% reduction in the number of foci. Average titer is listed above each group. Data in **B** and **C** are shown as mean ± SEM. LOD = 20 PFU. *P* values measured by Mann Whitney *U* test (BA.2.12.1 and B.1.351) or 1-way ANOVA followed by Tukey’s test for multiple comparisons (BA.5). *P < 0.05, **P < 0.01, ***P < 0.001, ****P < 0.0001.

Next, we investigated if these mice would still be protected against a lethal dose of a mouse-adapted virus, SARS2-N501Y_MA30_, 3 months after initial infection. Mice were reinfected with 5,000 PFU of SARS2-N501Y_MA30_, and weight loss was measured during acute infection (Figure 2A). There was complete protection against death with minimal weight loss in mice infected with any of the variants as compared with control groups (Figure 2B). Mice previously infected with B.1.351 experienced the least amount of weight loss while mice previously infected with XBB.1.5 experienced the most, at approximately 10% loss. The same sera that were used to measure neutralizing titers against the homologous virus were also tested against SARS2-N501Y_MA30_ to examine cross-reactivity. Sera were obtained prior to reinfection. We found strong neutralizing activity against SARS2-N501Y_MA30_ in mice infected with B.1.351, with no significant difference in titers between 20 and 60 days after initial infection (Figure 2C). In contrast, previous infection with BA.5 and BA.2.12.1 elicited much lower neutralizing titers, with neutralizing titers undetectable at 60 dpi with BA.2.12.1. Mice previously infected with XBB.1.5 did not mount a detectable level of neutralizing antibodies against SARS2-N501Y_MA30_ at any of the time points. These data indicate that there are factors contributing to protection against disease that did not involve neutralizing activity.

### XBB.1.5 induces a robust S protein–specific antibody response.

**Figure 3:**
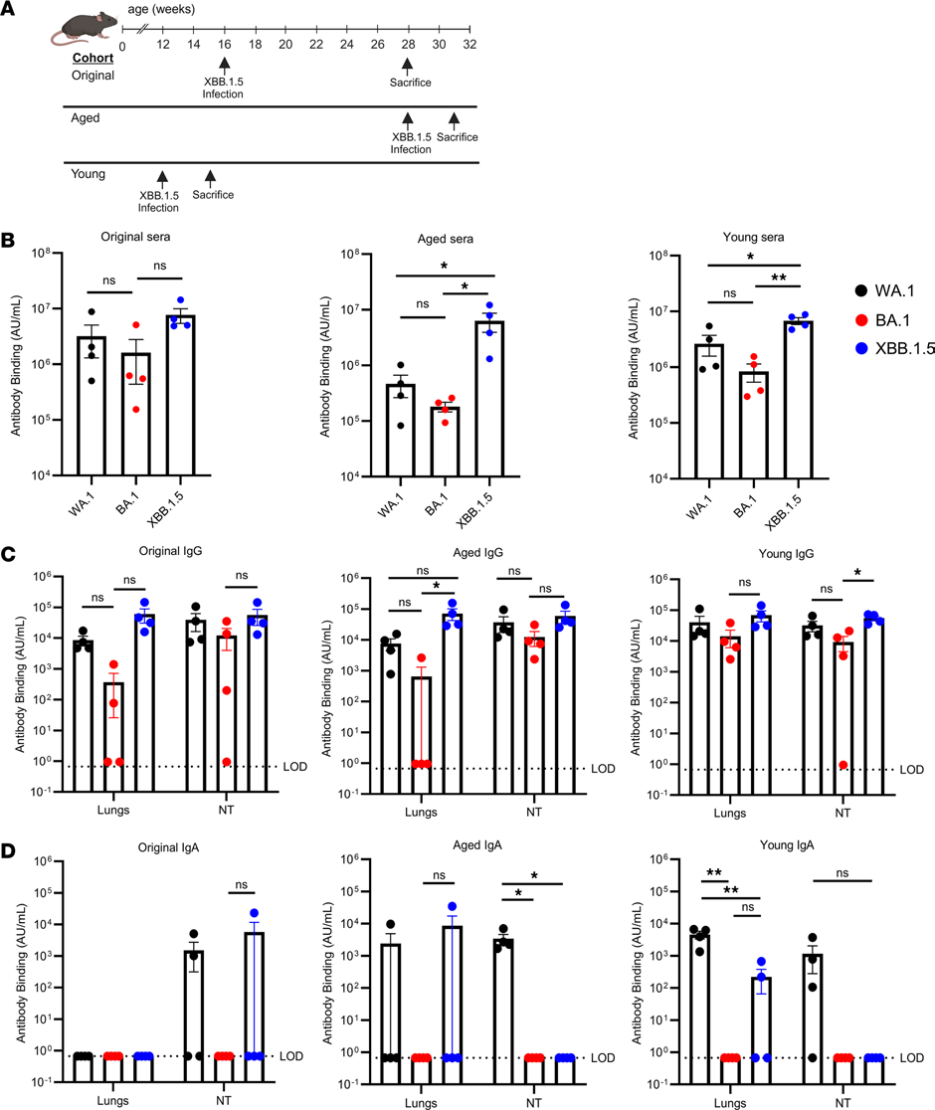
SARS-CoV-2–specific antibody binding in sera and nasal and lung tissues. Mice were infected intranasally with 10^5^ PFU of XBB.1.5. (**A**) Schematic detailing the timeline and cohorts used for the antibody binding experiments. Three different cohorts of mice were used in this experiment. (**B**–**D**) Sera and nasal turbinate and lung tissues were harvested at the indicated times for the measurement of (**B**) total antibody, (**C**) IgG, (**D**) and IgA binding to WA1, BA.1, and XBB.1.5 full-length spike proteins, as described in Methods. (**B**) Antibody binding in serum. (**C** and **D**) Nasal turbinate and lung tissue IgG (**C**) and IgA (**D**) binding. For each cohort, *n* = 4. Data are from 1 experiment. All results were obtained prior to reinfection with SARS2-N501Y_MA30_. *P* values were measured by 1-way ANOVA followed by Tukey’s test for multiple comparisons. Data in **B**–**D** are shown as mean ± SEM. LOD = 0.67. *P < 0.05, **P < 0.01.

Given the discrepancy between the clinical efficacy of prior infection with several variants of concern and the lack of a neutralizing antibody response, we next assessed whether broad spectrum S protein binding activity could be detected after XBB.1.5 infection. For this purpose, we measured IgG and IgA binding to SARS-CoV-2 spike proteins WA1, BA.1, and the homologous virus, XBB.1.5, in the sera and in nasal turbinate and lung homogenates. We examined 3 different groups of mice: the “original” group belonged to the same cohort of XBB.1.5-infected mice described above (Figure 1E and Figure 2, B and C). These mice were infected at 4 months of age and sacrificed 3 months later. Animals in the “aged” group were infected at 7 months of age with the XBB.1.5 variant and sacrificed 21 days later. Mice in the “young” group were infected at 12 weeks and sacrificed at 15 weeks of age, 21 days following XBB.1.5 infection (Figure 3A). The highest antibody binding in the sera was found to be against XBB.1.5 in each cohort, as expected (Figure 3B). Levels of binding to XBB.1.5 were also found to be comparable between the cohorts despite the differences in both age and duration after infection. In contrast, antibody responses to the WA.1 and BA.1 variants were lower, with the lowest responses observed 21 days after XBB.1.5 infection of 7-month-old mice. SARS-CoV-2 IgG responses in the lungs and nasal turbinates paralleled those observed in the sera, although antibody binding to all of the variant S proteins was more equivalent in the nasal turbinates. Again, the highest antibody binding was against XBB.1.5 while the lowest levels were against BA.1 (Figure 3C).

Contrary to these results, we detected SARS-CoV-2–specific IgA in only a fraction of homogenates. WA.1-specific IgA responses were detected in the nasal turbinates at 21 days after XBB.1.5 infection and in the lungs of young and aged mice (Figure 3D). Therefore, the complete protection of previously infected mice against subsequent lethal infection challenge despite the lack of detectable neutralizing antibodies against SARS2-N501Y_MA30_ is likely conferred by antibody-dependent processes, such as Fc-mediated responses in addition to T cells.

### Previously infected mice depleted of T cells have increased viral titers in nasal turbinates and, to a lesser extent, lungs upon reinfection.

**Figure 4:**
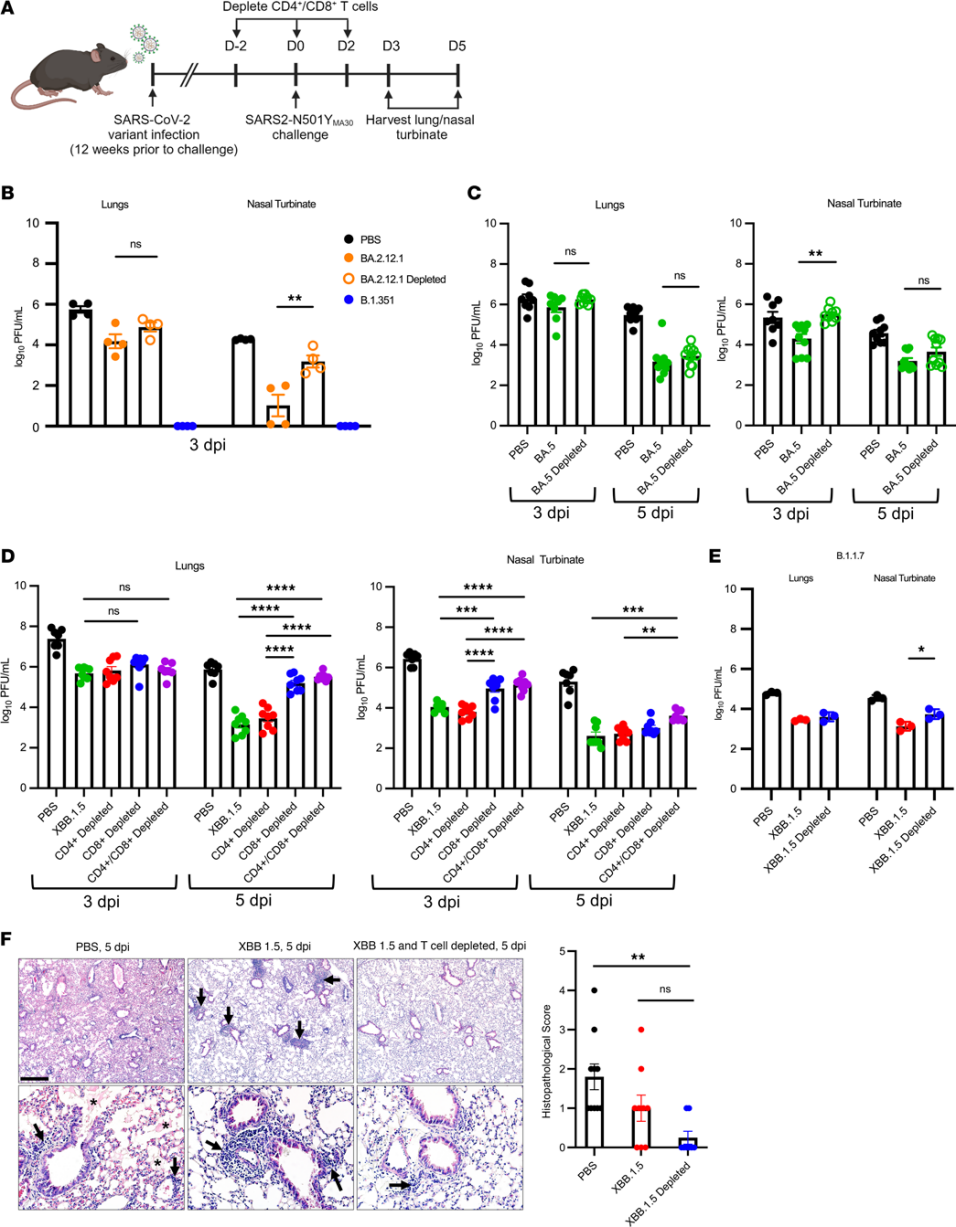
Effect of T cell depletion on kinetics of virus clearance. Four-month-old (XBB.1.5) or 6-month-old (other SARS-CoV-2 variants) C57BL/6 mice were infected with the indicated SARS-CoV-2 variant and challenged with SARS2-N501Y_MA30_ 3 months later. (**A**) Schematic detailing experimental timeline. CD4^+^/CD8^+^ T cells were depleted at the indicated time points. (**B**–**D**) Mice were initially infected with (**B**) BA.2.12.1, (**B**) B.1.351, (**C**) BA.5, or (**D**) XBB.1.5. PBS-treated mice were mock infected and then challenged with SARS2-N501Y_MA30_. (**B**) BA.2.12.1- and B.1.351-infected groups were non–T cell–depleted mice (*n* = 4/group). Data represent 1 experiment. (**C**) 3 dpi: PBS (*n* = 8), BA.5 (non–T cell depleted), (*n* = 8 lungs, *n* = 10 nasal turbinates (NT), and BA.5 depleted (*n* = 9). 5 dpi: PBS (*n* = 9), BA.5 (*n* = 10), and BA.5 depleted (*n* = 10). Data are from 2 independent experiments. (**D**) The XBB.1.5-infected group comprised non–T cell–depleted mice. Mice were CD4^+^ T cell, CD8^+^ T cell or CD4/CD8^+^ T cell depleted. Each group contained 7–8 mice, from 2 independent experiments. Data in **B**–**D** are shown as mean ± SEM. Each symbol represents data obtained from 1 mouse. (**E**) XBB.1.5-infected mice were challenged with the B.1.1.7 (α variant). Virus titers in the lungs and nasal turbinates were measured at 5 dpi. Each group contained 4 mice. Data are from 1 experiment. (**F**) Lung pathology of XBB.1.5, XBB.1.5 T cell depleted, and PBS-treated mice at 5 dpi. PBS (*n* = 10), XBB.1.5 (*n* = 9), and XBB.1.5 infected and T cell depleted (*n* = 8). Evidence of edema is denoted by asterisks, and cellular infiltrates are marked with arrows. Scale bar: 450 μm (top) and 90 μm (bottom). All *P* values were measured by 1-way ANOVA followed by Tukey’s test for multiple comparisons. *P < 0.05, **P < 0.01, ***P < 0.001, ****P < 0.0001.

CD4^+^ and CD8^+^ T cell responses have been implicated in protection against SARS-CoV-2 infection in several studies (14, 28–33). We next assessed the role of the T cell response in mice previously infected with the BA.2.12.1 or XBB.1.5 variants and challenged with SARS2-N501Y_MA30_. CD4^+^ and CD8^+^ T cells were depleted at the time of challenge. Depletion of T cells in the lung and nasal turbinate tissue was confirmed by flow cytometry, both in the vasculature (IV+) and the parenchyma (IV-) (Supplemental Figure 1, A–C; supplemental material available online with this article; https://doi.org/10.1172/jci.insight.184074DS1). Mice were then assessed for weight loss, and lungs and nasal tissue were collected for measurement of viral titers (Figure 2A). Depletion of T cells was not found to influence survival during lethal infection since mice had 0% mortality regardless of the variant used for initial infection (Figure 2B). Weight changes largely mimicked those of nondepleted mice both in XBB.1.5- and BA.2.12.1-infected mice. Next, viral loads in the lungs and in the nasal turbinates were measured at 3 and 5 dpi (Figure 4A). Within the cohort of mice previously infected with BA.2.12.1, we found no difference in lung virus titers between nondepleted (BA.2.12.1) and T cell–depleted (BA.2.12.1 depleted) mice (Figure 4B). However, in the nasal turbinates, T cell–depleted BA.2.12.1-infected mice were found to have significantly higher viral titers than mice in the nondepleted group. Mice infected with B.1.351, which had the highest neutralizing antibody titers (Figure 1B) did not have detectable levels of infectious virus in the lungs at 3 days after challenge with SARS2-N501Y_MA30_ (Figure 4B). Since neutralizing antibody responses persisted in mice infected with B.1.351, T cell depletion assays were not performed using this virus. Mice previously infected with BA.5 virus showed a similar phenotype to BA.2.12.1-infected mice; T cell–depleted mice and nondepleted mice had no differences in viral lung titers at 3 and 5 dpi (Figure 4C). Again, there were significantly higher viral titers in the nasal turbinates of T cell–depleted mice compared with the nondepleted group at 3 dpi.

To further explore the effect of T cells upon a second exposure to SARS-CoV-2, mice in the XBB.1.5 cohort were treated with α-CD4^+^ or α-CD8^+^ antibody, with both antibodies or not depleted at all prior to SARS2-N501Y_MA30_ challenge. After prior infection with XBB.1.5 and T cell depletion, virus clearance in the lungs was diminished at 5 dpi. Mice that were depleted of CD8^+^ or doubly depleted of CD4^+^ and CD8^+^ T cells exhibited higher SARS2-N501Y_MA30_ lung titers than mice that were CD4^+^ T cell or mock depleted (Figure 4D). Of note, BA.2.12.1-infected mice also exhibited a lack of neutralizing antibodies against SARS2-N501Y_MA30_ at 60 dpi, and while the differences in lung titers between BA.2.12.1 T cell–depleted mice and BA.2.12.1 mice were not found to be significant, those in BA.2.12.1-infected mice trended lower than those in the depleted group (Figure 4B).

We detected higher viral titers in the nasal turbinates of mice previously infected with XBB.1.5 after CD4^+^/CD8^+^ or CD8^+^ T cell depletion compared with nondepleted mice (Figure 4D). This was most pronounced at 3 dpi, where titers in CD4^+^ T cell–depleted mice closely resembled mice that were not depleted while titers in CD8^+^ T cell–depleted mice were similar to those detected in doubly depleted mice. As in XBB.1.5-infected mice, greater differences were observed in the nasal turbinates after T cell depletion in BA.2.12.1 mice (Figure 4B). Pathological examination of the lungs of mice previously infected with XBB.1.5 and then reinfected with SARS2-N501Y_MA30_ showed high levels of edema and cellular infiltrates in the PBS group at 5 dpi (Figure 4F). In contrast, there was minimal evidence of tissue damage in both the nondepleted and T cell–depleted groups after infection with XBB.1.5. However, T cell–depleted mice had decreased numbers of cellular infiltrates present as compared with mice in which these cells were not depleted, consistent with the absence of T cells.

To determine whether these results obtained after infection with SARS2-N501Y_MA30_ (derived from the ancestral Wuhan-1 strain) were also observed after challenge with another early appearing variant, mice that were previously infected with XBB.1.5 were reinfected with the B.1.1.7 variant. Viral titers in the nasal turbinate of depleted mice were significantly higher at 5 dpi than those of mice that were not T cell depleted (Figure 4E). Together, these results suggest that there is a protective role for memory CD8^+^ T cells in the response to subsequent virus infection but also that they do not appear to be necessary for protection from clinical disease in mice.

### Antigen-experienced tissue-resident memory T cells are increased in the nasal cavity of previously infected mice upon reinfection.

**Figure 5:**
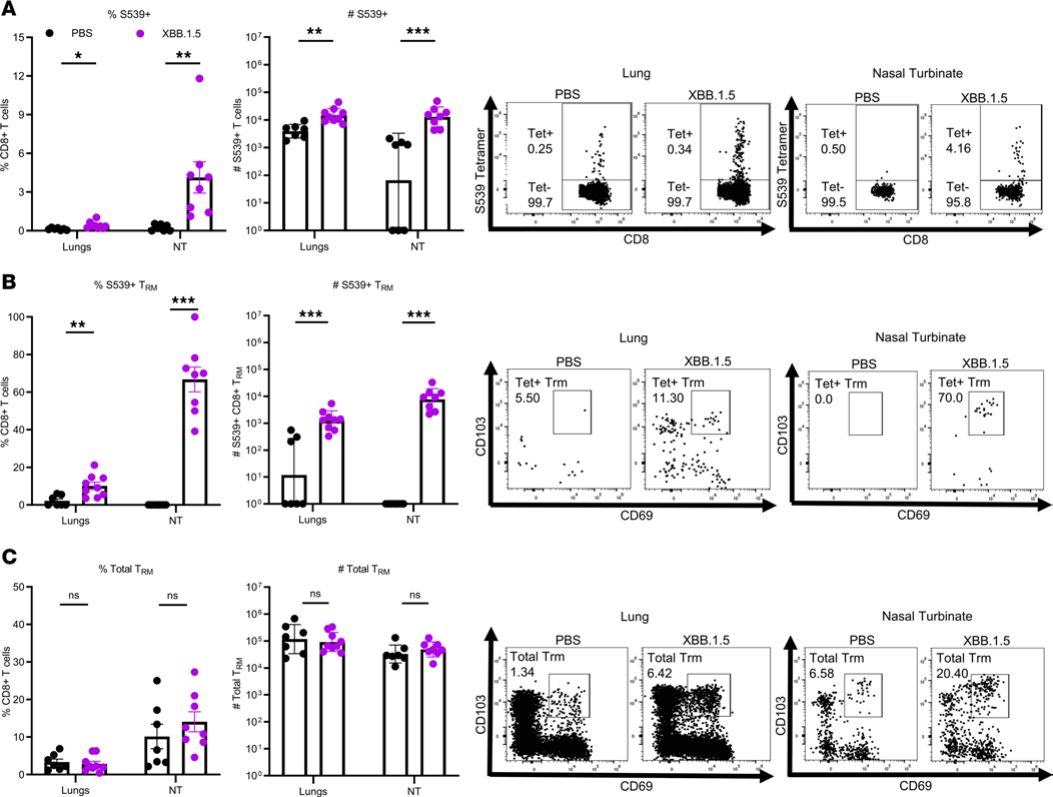
Memory T cell characterization at 3 days after challenge. Mice were challenged with SARS2-N501Y_MA30_ 3 months after XBB.1.5 infection. Mice were briefly treated with PerCP-Cy5.5–conjugated anti-Thy1.2 to label cells in the vasculature, as described in the Methods. Lungs and nasal turbinates were harvested for class I tetramer staining 3 days after reinfection and Thy1.2^–^ cells were analyzed by flow cytometry. PBS lungs (*n* = 8), PBS nasal turbinates (NT) (*n* = 7). XBB.1.5 lungs (*n* = 9), XBB.1.5 NT (*n* = 8). Data represent 2 independent experiments. *P* values were measured by Mann-Whitney *U* test. (**A**) Frequency (left) and number (right) of S539 tetramer^+^ T cells gated on the CD8^+^ T cell population. Representative plots for lungs (left) and nasal turbinates (right) are shown. (**B**) Frequency (left) and number (right) of virus-specific Trm cells gated on S539 tetramer^+^ T cells. Representative plots for lungs (left) and nasal turbinates (right) are shown. (**C**) Frequency (left) and number (right) of total Trm cells gated on the CD8^+^ T cell population. Representative plots for lungs (left) and nasal turbinates (right) are shown. Data for Trm frequency are shown as mean ± SEM. Data for Trm number are shown as geometric mean ± geometric SD. *P < 0.05, **P < 0.01, ***P < 0.001.

These data suggest an important role for virus-specific memory CD8^+^ T cells in the nasal turbinates and, to a lesser extent, in the lungs in reducing virus burden. To assess whether these cells can be detected at these sites of infection, we infected mice with XBB.1.5 and then subsequently reinfected them with 2,000 PFU SARS2-N501Y_MA30_ 3 months later. Lungs and nasal turbinates were harvested 3 days after challenge. Virus-specific CD8^+^ T cells were assessed by MHC class I (H2-K^b^) S539 tetramer staining. There was a significant increase in the frequency and numbers of tetramer S539^+^ cells in the nasal turbinates and, to a lesser extent, in the lungs of SARS2-N501Y_MA30_-challenged mice that had been previously infected with XBB.1.5 (Figure 5A). We additionally characterized this tetramer S539^+^ population in the nasal turbinates and found that it largely consisted of tissue-resident memory T (Trm) cells identified by CD69^+^CD103^+^ staining. Gating strategy shown in Supplemental Figure 2. Given that these cells expressed Trm cell markers, it is not likely that they originated in extranasal tissue. In addition, since mice were analyzed at 3 days after challenge, it is unlikely that they originated de novo from naive T cell populations. Previously infected mice had a significant increase in tetramer S539^+^ CD8^+^ Trm cell frequency and numbers in the nasal turbinates compared with the PBS group, which was also significant in the lungs, though less pronounced (Figure 5B). Finally, the overall Trm cell population between the PBS-treated mice and the mice previously infected with XBB.1.5 did not differ in both the nasal turbinate and lung tissues (Figure 5C).

## Discussion

As SARS-CoV-2 becomes endemic, the virus continued to mutate rapidly in response to immunity induced by vaccines and prior infections to generate antigenically distant variants replacing earlier strains. In response, vaccines were updated because early formulations were less effective against these new variants in terms of inducing neutralizing antibodies. One consequence of the changes in viruses and vaccines is that unvaccinated, uninfected individuals, including young children will not be exposed to past variants and, therefore, may develop no immunity against such previous variants that are no longer circulating. Reemergence of past variants may pose significant threats to future generations who have never been exposed to these original variants, as previously demonstrated for influenza A virus infection. During the 2009 H1N1 outbreak, young people were disproportionately affected by infection, while those born before 1957 experienced the lowest rates of morbidity (34–37). This was largely attributed to the preexisting cross-reactive H1N1 antibodies and cell-mediated immunity acquired from childhood infection (38, 39).

Here, we showed that previous infection with B.1.351/Omicron variants protected against severe disease in mice after challenge with SARS2-N501Y_MA30_, a mouse-adapted SARS-CoV-2 strain closely resembling the ancestral strain, suggesting that infection with the more recent SARS-CoV-2 variants induced cross-protection against early variants. Similarly, immunization with mRNA vaccines encoding spike protein of the ancestral Wuhan-1 strain or prior infection with early SARS-CoV-2 variants protected against severe disease after infection with B.1.351/Omicron variants (40, 41). Together, these data indicate that immunity induced by vaccination or infection with antigenically distant SARS-CoV-2 strains are sufficient to cross-protect against severe disease after heterologous SARS-CoV-2 infection.

Levels of neutralizing antibody were previously identified as correlates of protection in the period when original circulating variants were circulating (42, 43). However, neutralizing antibodies are not the sole mechanism of protection since vaccinated humans are protected against severe disease and hospitalization but not infection after exposure to Omicron variants. Similarly, we observed that mice infected with B.1.351/Omicron variants were almost completely protected against subsequent challenge with a lethal dose of SARS2-N510Y_MA30_ (Figure 2B). This phenomenon was independent of the immunizing variant, even in conditions where the levels of neutralizing titers to homologous or heterologous virus were low (XBB.1.5-infected mice, Figure 1E and Figure 2C), suggesting that other immune functions contributed to protection. One of these factors is the T cell response. We observed that depletion of T cells resulted in delayed virus clearance in the nasal turbinates and, to a lesser extent, the lungs (Figure 4C), confirming the role of T cells in controlling infection. Consistent with the role of prior infection in the induction of this T cell response, we showed that many of these T cells were resident memory T cells (Figure 5). Similarly, memory CD4^+^ and CD8^+^ T are rapidly induced following SARS-CoV-2 infection of vaccinated individuals (44). In addition, studies have shown that repeated vaccination or infection with mismatched SARS-CoV-2 strains often results in immunological imprinting (45, 46), therefore potentially contributing to reduced vaccine efficacy or protection. However, the T cell response was much less affected by imprinting, as T cell epitopes were only modestly changed in new SARS-CoV-2 variants (47). T cell targets are also more diverse with epitopes located in multiple viral proteins in addition to S protein. Therefore, vaccine strategies specifically boosting T cell responses as previously suggested (48) will help compensate for immunological imprinting and limit the emergence of escape mutants.

While our data suggest that virus-specific Trm cells contributed to protection in both the lungs and nasal turbinates (Figures 4 and 5), T cells were required for virus elimination to a greater extent in the latter than in the lungs (Figure 4). Although we showed that the frequency and number of Trm cells were diminished after depletion (Supplemental Figure 1C), a limitation of our study is that Trm cells could not be selectively depleted. Therefore, determining the specific role of Trm cells in mediating protection warrants further investigation. In addition, another limitation is that we used inbred mouse strains and mouse-adapted virus, which does not reflect the diversity of immune responses in humans. Furthermore, T cell depletion only resulted in increased viral burden without detectable changes in clinical disease (Figures 2 and 4). Protection could result from the presence of nonneutralizing SARS-CoV-2–specific IgGs in the lungs and nasal turbinates (49) (Figure 3). Other studies identified virus-specific IgA responses in the respiratory mucosa and the presence of long-lasting memory B cells after infection and vaccination (50–52), which also likely contributed to protection. We were not able to detect significant IgA antibodies in the nasal turbinates of infected mice, possibly because IgA was present at lower levels than IgG or IgA assays were less sensitive.

Overall, we showed that infection in mice with recent SARS-CoV-2 variants protected against challenge with early variants, consistent with key roles for neutralizing antibody-independent functions in protection. Moreover, we demonstrated an important role for T cell–mediated protection in the upper airway, which will inform the design of next generation of mucosal vaccines against coronavirus infections.

## Methods

### Sex as a biological variable.

Our preliminary study used infected male and female mice. Nearly identical results were obtained. We used only female mice for subsequent studies because they were slightly more resistant to SARS-CoV-2 infection.

### Cells and virus.

All SARS-CoV-2 variants were obtained from BEI Resources: B.1.351 (β variant, NR-55282), BA.2.12.1 (NR-56781), BA.5 (NR-58616), XBB.1.5 (NR-59104), and B.1.1.7 (α variant, NR-54971). Mouse-adapted SARS2-N501Y_MA30_ was generated as described previously (19). B.1.1.7, B.1.351, and SARS2-N501Y_MA30_ were propagated in Calu-3 2B4 cells while Omicron variants BA.2.12.1, BA.5, and XBB.1.5 were propagated in Vero-TMPRSS2 cells. Calu-3 2B4 cells were obtained from Chien-Te Kent Tseng at the University of Texas Medical Branch in Galveston (Galveston, Texas, USA) and were grown in DMEM (GIBCO) supplemented with 20% FBS. Vero-TMPRSS2 cells were obtained from Michael Diamond (Washington University, St. Louis, Missouri, United States.) and were grown in DMEM supplemented with 10% FBS and 5 μg/mL blasticidin. Vero hACE2-TMPRSS2 cells, obtained from Michael Diamond, were used for the foci reduction neutralization test (FRNT_50_) and focus-forming assay experiments (see below) and were cultured in DMEM supplemented with 10% FBS, 1 M HEPES (GIBCO), and 10 μg/mL puromycin.

### SARS-CoV-2 variant infection and SARS2-N501Y_MA30_ or B.1.1.7 challenge in mice.

Female C57BL/6 mice were purchased from Charles River Laboratories. Mice were anesthetized with ketamine-xylazine and infected intranasally with 10^5^ PFU of the indicated virus. In some experiments, infected mice were monitored daily for 3–4 months before subsequent challenge with 5,000 PFU of SARS2-N501Y_MA30_ or 10^5^ PFU B.1.1.7. After reinfection, mice were monitored for weight loss and clinical disease. In other experiments (Figure 3), 12-week-old (young) and 7-month-old (aged) mice were infected intranasally with 10^5^ PFU of XBB.1.5 and monitored daily for 21 days before sacrifice. All experiments with SARS-CoV-2 were performed in a biosafety level 3 laboratory at the University of Iowa.

### Viral titers.

Infected, challenged mice were sacrificed at 3 or 5 dpi and perfused intracardially with 10 mL PBS. Lungs and nasal turbinates were harvested and homogenized in 1 mL PBS. Samples were aliquoted and stored at –80°C. Titers were measured by focus-forming assays using Vero hACE2-TMPRSS2 cells. Cells were seeded in 96-well plates and inoculated in 10-fold serial dilutions with lung or nasal turbinate homogenates for 1 hour at 37°C, 5% CO_2_, gently rocking every 10 minutes. Then, the inoculum was removed, and cells were overlaid with 1:1 mixture of 2.4% carboxymethylcellulose and DMEM containing 4% FBS. Cells were stained and foci were visualized as detailed below.

### FRNT_50_.

FRNTs were used to measure the neutralizing antibody activity against the SARS-CoV-2 variants and mouse-adapted SARS2-N501Y_MA30_ virus. Mice were anesthetized by intraperitoneal injection of ketamine-xylazine. Blood was collected through retro-orbital bleed with a capillary tube (Fisher Scientific). Blood was allowed to clot at room temperature for 60 minutes before centrifugation. Sera were removed into a new tube and stored at –20°C. Serial dilutions of the sera were incubated with an equal volume of 90–100 foci of the indicated virus at 37°C for 1 hour. Subsequently, 50 μL of the mixture was added to confluent Vero hACE2-TMPRSS2 cells in 96-well plates and incubated at 37°C, 5% CO_2_ for 1 hour. After incubation, the inoculum was removed and 100 μL overlay (1:1 mixture of 2.4% carboxymethylcellulose and DMEM containing 4% FBS) was applied to each well. Plates were incubated at 37°C, 5% CO_2_ for 24 hours. After, cells were fixed with 200 μL of 4% paraformaldehyde for 1 hour at room temperature. Fixative was removed, and cells were washed and then permeabilized with 0.75% Triton-X100 for 20 minutes, followed by incubation with primary rabbit monoclonal α-SARS-CoV nucleocapsid antibody (1:1,000 for 1 hour at 37°C, Sino Biological). Cells were then washed and incubated in secondary rabbit HRP-conjugated IgG antibody (1:500 for 1 hour at 37°C, Biolegend). Foci were visualized by addition of KPL TrueBlue peroxidase substrate (Sera-care) for 10 minutes at room temperature. The log antibody concentration was plotted against the percentage of inhibition of each concentration, and the IC_50_ was calculated using a nonlinear variable slope equation: *Y* = 100/(1 + 10^((logIC50^^–^^X)^^×^^HillSlope)^.

### Histopathology.

Mice were anesthetized by intraperitoneal injection of ketamine-xylazine and perfused with 10 mL PBS. Tissues were fixed in zinc formalin and then embedded in paraffin. For routine histology, tissue sections (~4 μm) were stained with H&E and examined by a boarded veterinary pathologist. For experiments in which mice were infected with SARS2-N501Y_MA30_, lung tissues were examined in a postexamination method of masking to group assignment (53). Lung edema and cellular infiltrate scores were evaluated based on extent of distribution as previously performed (54). High-resolution images were taken using a BX53 microscope, DP73 digital camera, and Cell Sens Dimension software (Olympus).

### Antibody binding assay.

Blood sera and tissue homogenates of lungs and nasal turbinates from XBB.1.5-infected mice were assessed for the presence of IgG and IgA antibodies targeting the SARS-CoV-2 WA1, BA.1, and XBB.1.5 spike proteins using the V-PLEX SARS-CoV-2 Panel 34 (Mouse IgG) kit and V-PLEX SARS-CoV-2 Panel 34 (Mouse IgA) kit (Meso Scale Discovery, K15696U-4) following the instructions provided by the manufacturer (55). The only difference between the Wuhan-1 strain, used in most experiments herein, and the WA1 strain is 1 amino acid change in ORF8 and 2 silent alterations (in ORF1a and ORF1b). Initially, antigen-specific plates were prepared by blocking with MSD blocker at room temperature and shaking at 700 rpm for 30 minutes. The samples were then diluted 1:500, 1:5,000, and 1:50,000 and placed on the plates for 2 hours at room temperature. Subsequently, SULFO-TAG–conjugated Goat anti-Mouse IgG or IgA antibody was added to their respective plates (Meso Scale Discovery). Next, the plates were rinsed with 1X MSD wash buffer, followed by addition of MSD Gold Read Buffer B to each well. Plates were washed 3 times with wash buffer after each stage. Optical densities were measured using an MSD plate reader, and the data were analyzed with Discovery Workbench software, version 4.0. Antibody levels were reported in arbitrary units per mL specific to SARS-CoV-2.

### T cell depletion.

Mice were depleted of CD4^+^ and/or CD8^+^ T cells by intraperitoneal injection of 250 μg α-CD4^+^ mouse antibody (clone GK1.5, Leinco Technologies) and/or 250 μg α-CD8^+^ mouse antibody (clone 2.43, Leinco Technologies) in 250 μL. Mice received antibody at days –2, 0, and +2 relative to challenge with SARS2-N501Y_MA30_.

### Tetramer staining.

Previously infected and PBS-treated mice were infected with 2,000 PFU of SARS2-N501Y_MA30_ and sacrificed at 3 dpi. Lungs and nasal turbinates were perfused with 10 mL PBS and then harvested. Preparation of cells was performed as previously described (56). In short, tissues were minced and then digested in 1 mg/mL collagenase D (Roche Diagnostics) and 0.1 mg/mL DNase I (Roche Diagnostics) and nutated at 37°C for 1 hour. Tissues were then filtered twice through 70 μM cell strainers and washed before counting and subsequent staining. Virus-specific T cells were detected using APC-conjugated H2-K^b^ S539 tetramers obtained from the NIH Tetramer Facility (National Institute of Allergy and Infectious Disease MHC Tetramer Core Facility). Cells were stained with 5 μg/mL S539 tetramer for 30 minutes at 4°C. The following antibodies were used: CD16/CD32 (2.4G2), LIVE/DEAD fixable violet stain (Thermo Fisher), Super Bright Complete Staining Buffer (eBioscience), Thy 1.2 (30-H12, Biolegend), CD45 (30-F11, Biosciences), CD3 (145-2C11, Invitrogen), CD4 (GK1.5, BD Horizon), CD8a (53-6.7, BD Biosciences), CD11a (2D7, BD Biosciences), CD49a (Ha31/8, BD Biosciences), CD69 (H1.2F3, BD Biosciences), and CD103 (M290, BD Horizon). Data were collected using a Cytek Aurora spectral flow cytometer.

### Intravenous exclusion.

Prior to sacrifice (Figure 5), mice were treated with 2 μg PerCP-Cy5.5–conjugated Thy1.2 antibody by intravenous injection for 5 minutes. Mice were then processed as described above. Samples were analyzed by flow cytometry; Thy1.2^+^ populations were denoted as IV+ and Thy1.2^–^ populations were as denoted IV–. All data presented in Figure 5 are analyzed on the Thy1.2^–^ population.

### Statistics.

Statistical analyses were performed using Graph Pad Prism version 10.2.3 software. Statistical significance was determined by Mann Whitney *U* test, 1-way ANOVA with Tukey’s test for multiple comparisons, or log-rank followed by Bonferroni’s correction for multiple comparisons. A *P* value of less than 0.05 was considered statistically significant.

### Study approval.

All animal studies were approved by the University of Iowa Animal Care and Use Committee and meet stipulations of the *Guide for the Care and Use of Laboratory Animals* (National Academies Press, 2011).

### Data availability.

All the data are included in the manuscript or available from the corresponding authors upon request. Values for all data points in graphs are reported in the Supporting Data Values file.

## Author contributions

AO, LYRW, AS, MSS, and SP conceived the work and designed the experiments. AO, LYRW, MK, and AKV acquired the data. AO, LYRW, AS, DKM, MK, MSS, and SP analyzed the data. AO, LYRW, and SP wrote the manuscript.

## Supplementary Material

Supplemental data

Supporting data values

## Version 1 10/15/2024

In-Press Preview

## Version 2 12/06/2024

Electronic publication

## References

[1] Markov PV (2023). The evolution of SARS-CoV-2. Nat Rev Microbiol.

[2] Carabelli AM (2023). SARS-CoV-2 variant biology: immune escape, transmission and fitness. Nat Rev Microbiol.

[3] Mannar D (2024). Altered receptor binding, antibody evasion and retention of T cell recognition by the SARS-CoV-2 XBB.1.5 spike protein. Nat Commun.

[4] Tegally H (2022). Emergence of SARS-CoV-2 Omicron lineages BA.4 and BA.5 in South Africa. Nat Med.

[5] Weigang S (2021). Within-host evolution of SARS-CoV-2 in an immunosuppressed COVID-19 patient as a source of immune escape variants. Nat Commun.

[6] Clark SA (2021). SARS-CoV-2 evolution in an immunocompromised host reveals shared neutralization escape mechanisms. Cell.

[7] Marques AD (2024). SARS-CoV-2 evolution during prolonged infection in immunocompromised patients. mBio.

[8] Goldblatt D (2022). Correlates of protection against SARS-CoV-2 infection and COVID-19 disease. Immunol Rev.

[9] Liu J (2021). BNT162b2-elicited neutralization of B.1.617 and other SARS-CoV-2 variants. Nature.

[10] Nemet I (2022). Third BNT162b2 vaccination neutralization of SARS-CoV-2 Omicron infection. N Engl J Med.

[11] Zou Jing (2023). Neutralization of BA.4-BA.5, BA.4.6, BA.2.75.2, BQ.1.1, and XBB.1 with Bivalent vaccine. N Engl J Med.

[12] (2022). Durability of booster mRNA vaccine against SARS-CoV-2 BA.2.12.1, BA.4, and BA.5 subvariants. N Engl J Med.

[13] Canetti M (2022). Six-month follow-up after a fourth BNT162b2 vaccine dose. N Engl J Med.

[14] Ying B (2024). Mucosal vaccine-induced cross-reactive CD8^+^ T cells protect against SARS-CoV-2 XBB.1.5 respiratory tract infection. Nat Immunol.

[15] Lee A (2024). BCG vaccination stimulates integrated organ immunity by feedback of the adaptive immune response to imprint prolonged innate antiviral resistance. Nat Immunol.

[16] Li Y (2024). SARS-CoV-2 viral clearance and evolution varies by type and severity of immunodeficiency. Sci Transl Med.

[17] Hoffmann M (2023). Omicron subvariant BA.5 efficiently infects lung cells. Nat Commun.

[18] Baz M (2022). SARS-CoV-2 Omicron BA.1 challenge after ancestral or Delta infection in mice. Emerg Infect Dis.

[19] Wong L-YR (2022). Eicosanoid signalling blockade protects middle-aged mice from severe COVID-19. Nature.

[20] Pérez-Alós L (2022). Modeling of waning immunity after SARS-CoV-2 vaccination and influencing factors. Nat Commun.

[21] Gaebler C (2021). Evolution of antibody immunity to SARS-CoV-2. Nature.

[22] Levin Einav G (2021). Waning immune humoral response to BNT162b2 Covid-19 vaccine over 6 months. N Engl J Med.

[23] Seow J (2020). Longitudinal observation and decline of neutralizing antibody responses in the three months following SARS-CoV-2 infection in humans. Nat Microbiol.

[24] Halfmann PJ (2022). SARS-CoV-2 Omicron virus causes attenuated disease in mice and hamsters. Nature.

[25] Rizvi ZA (2023). Mendelian randomization analyses explore the relationship between cathepsins and lung cancer. Commun Biol.

[26] Uraki R (2022). Characterization and antiviral susceptibility of SARS-CoV-2 Omicron BA.2. Nature.

[27] Srivastava K (2024). SARS-CoV-2-infection- and vaccine-induced antibody responses are long lasting with an initial waning phase followed by a stabilization phase. Immunity.

[28] Liu J (2022). CD8 T cells contribute to vaccine protection against SARS-CoV-2 in macaques. Sci Immunol.

[29] Chandrashekar A (2022). Vaccine protection against the SARS-CoV-2 Omicron variant in macaques. Cell.

[30] Liu J (2022). Vaccines elicit highly conserved cellular immunity to SARS-CoV-2 Omicron. Nature.

[31] Kar M (2024). CD4+ and CD8+ T cells are required to prevent SARS-CoV-2 persistence in the nasal compartment. Sci Adv.

[32] Lieber CM (2024). Efficacy of late-onset antiviral treatment in immune-compromised hosts with persistent SARS-CoV-2 infection. J Virol.

[33] Fumagalli V (2024). Antibody-independent protection against heterologous SARS-CoV-2 challenge conferred by prior infection or vaccination. Nat Immunol.

[34] Fisman David N (2009). Older age and a reduced likelihood of 2009 H1N1 virus infection. N Engl J Med.

[35] Xu R (2010). Structural basis of preexisting immunity to the 2009 H1N1 pandemic influenza virus. Science.

[36] Jain S (2009). Hospitalized patients with 2009 H1N1 influenza in the United States, April–June 2009. N Engl J Med.

[37] Chowell Gerardo (2009). Severe respiratory disease concurrent with the circulation of H1N1 influenza. N Engl J Med.

[38] Greenbaum JA (2009). Pre-existing immunity against swine-origin H1N1 influenza viruses in the general human population. Proc Natl Acad Sci U S A.

[39] Hancock K (2009). Cross-reactive antibody responses to the 2009 pandemic H1N1 influenza virus. N Engl J Med.

[40] Ying B (2022). Boosting with variant-matched or historical mRNA vaccines protects against Omicron infection in mice. Cell.

[41] Chin ET (2022). Protection against Omicron from vaccination and previous infection in a prison system. N Engl J Med.

[42] Khoury DS (2021). Neutralizing antibody levels are highly predictive of immune protection from symptomatic SARS-CoV-2 infection. Nat Med.

[43] Feng S (2021). Correlates of protection against symptomatic and asymptomatic SARS-CoV-2 infection. Nat Med.

[44] Painter MM (2023). Prior vaccination promotes early activation of memory T cells and enhances immune responses during SARS-CoV-2 breakthrough infection. Nat Immunol.

[45] Johnston TS (2024). Immunological imprinting shapes the specificity of human antibody responses against SARS-CoV-2 variants. Immunity.

[46] Park Y-J (2022). Imprinted antibody responses against SARS-CoV-2 Omicron sublineages. Science.

[47] Choi SJ (2022). T cell epitopes in SARS-CoV-2 proteins are substantially conserved in the Omicron variant. Cell Mol Immunol.

[48] Moss P (2022). The T cell immune response against SARS-CoV-2. Nat Immunol.

[49] Tan TJC (2024). Evidence of antigenic drift in the fusion machinery core of SARS-CoV-2 spike. Proc Natl Acad Sci U S A.

[50] Havervall S (2022). Anti-spike mucosal IgA protection against SARS-CoV-2 Omicron infection. N Engl J Med.

[51] Turner JS (2021). SARS-CoV-2 infection induces long-lived bone marrow plasma cells in humans. Nature.

[52] Turner JS (2021). SARS-CoV-2 mRNA vaccines induce persistent human germinal centre responses. Nature.

[53] Meyerholz DK, Beck AP (2018). Principles and approaches for reproducible scoring of tissue stains in research. Lab Invest.

[54] Zheng J (2021). COVID-19 treatments and pathogenesis including anosmia in K18-hACE2 mice. Nature.

[55] Edara VV (2021). Infection- and vaccine-induced antibody binding and neutralization of the B.1.351 SARS-CoV-2 variant. Cell Host Microbe.

[56] Zhao J (2009). De novo recruitment of antigen-experienced and naive T cells contributes to the long-term maintenance of antiviral T cell populations in the persistently infected central nervous system. J Immunol.

