# Supplementary figures and images for: Tissue-resident memory T cells contribute to protection against heterologous SARS-CoV-2 challenge
Source: JCI Insight. 2024 Dec 6;9(23):e184074. doi: 10.1172/jci.insight.184074 (PMC11623939; doi:10.1172/jci.insight.184074)

A

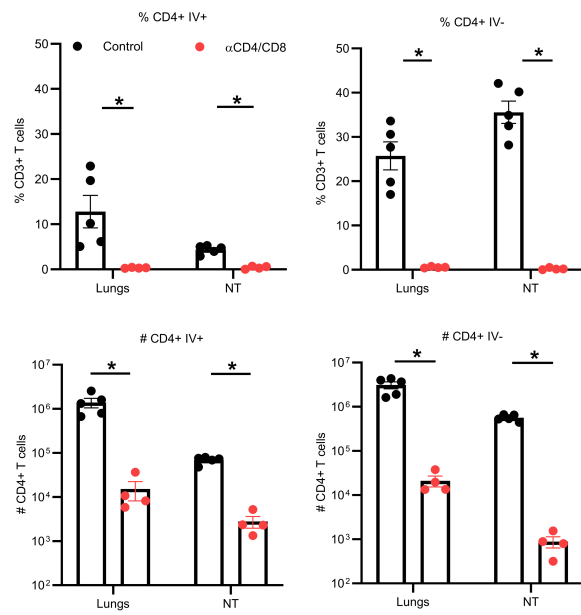

B

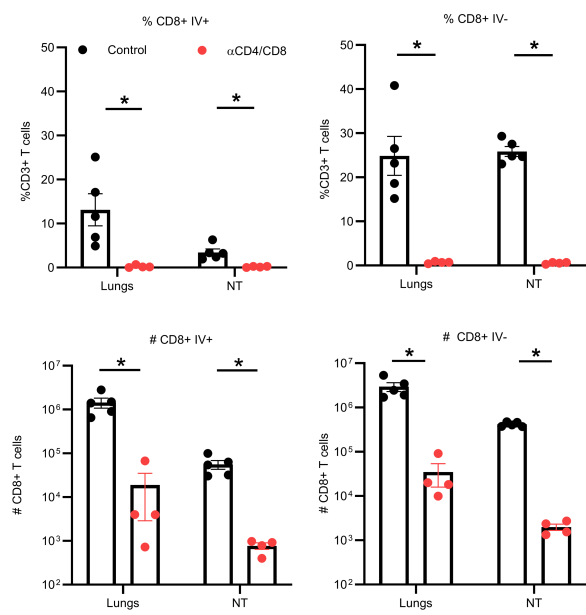

C

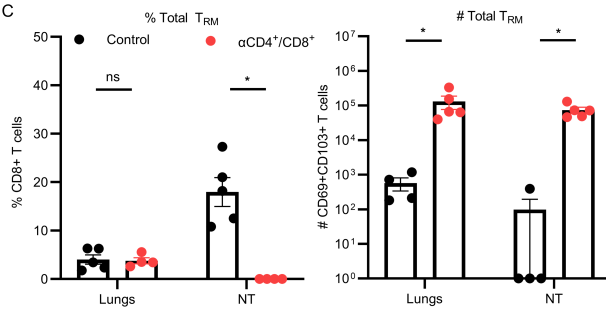

**A**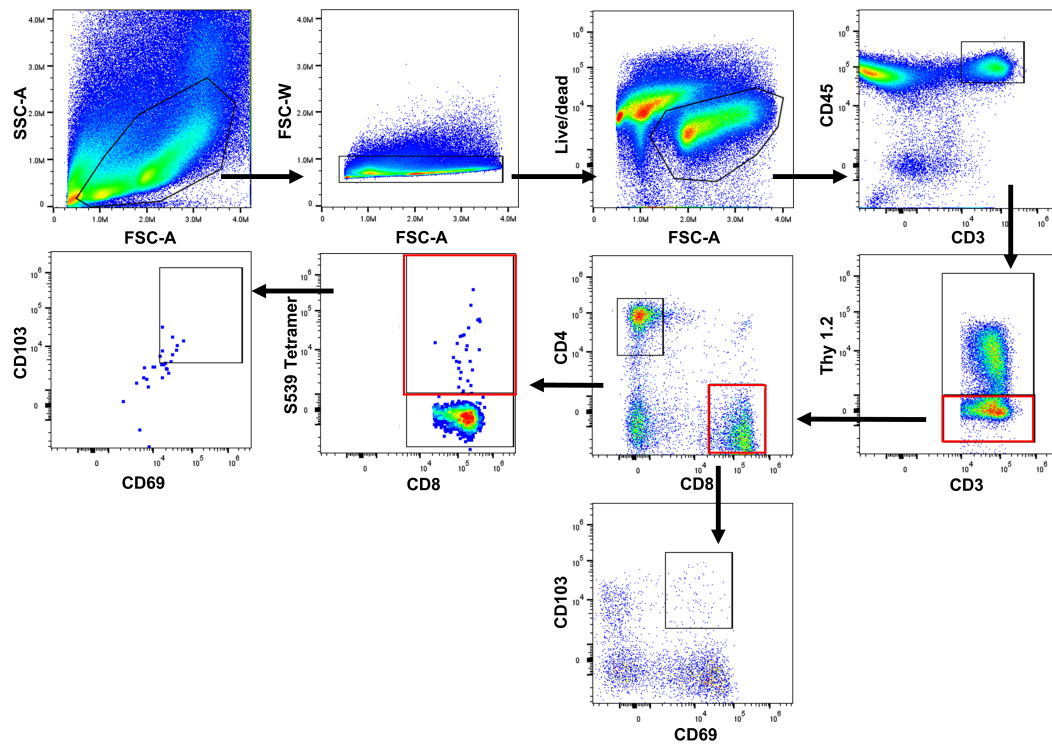**B**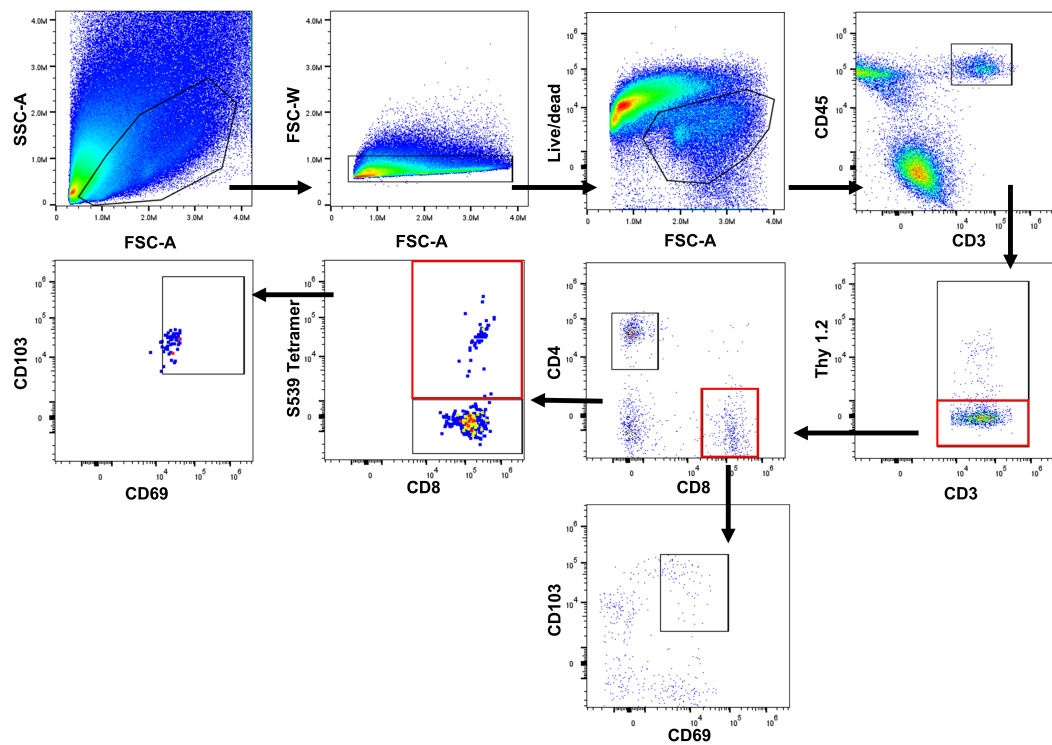

**Supplementary Figure 2**

Supplement: Supplemental data [file jciinsight-9-184074-s117.pdf]
